# Supplementary material for: Mechanically compliant and cost-effective 1.4Li2O-0.75ZrCl4-0.25AlCl3 solid electrolyte for all-solid-state batteries with improved cycling stability
Source: Nat Commun. 2026 Jan 8;17:1474. doi: 10.1038/s41467-025-68210-5 (PMC12886765; doi:10.1038/s41467-025-68210-5)
Supplement: Supplementary file 1 — Supplementary Information [file 41467_2025_68210_MOESM1_ESM.pdf]

Supplementary Information

Mechanically compliant and cost-effective $1.4Li_2O$-$0.75ZrCl_4$-$0.25AlCl_3$ solid electrolyte for all-solid-state batteries with improved cycling stability

Lv Hu[1,2†], Yaolong He[3†], Dong Wang[4], Wanxia Li[5], Jingming Yao[6], Xiaolong Zhang[7], Jinfeng Zhu[8], Huaican Chen[9], Wen Yin[9], Yanru Wang[10], Kejun Yan[11], Jinzhu Wang[1], Hui Li[1], Fang Chen[1], Yating Liu[1], Junqi Lai[12], Qi Chen[12], Jie Ma[8], Shuhong Jiao[5], Guorui Wang[4], Siqi Shi[3*], Liwei Chen[13,14,15], Jianyu Huang[6], and Cheng Ma[1,2,16*]

[1]Hefei National Research Center for Physical Sciences at the Microscale, University of Science and Technology of China, Hefei, Anhui 230026, China

[2]Gu-ning Aevum New Energy Technology Co., Ltd., Hefei, Anhui 230088, China

[3]State Key Laboratory of Materials for Advanced Nuclear Energy, Shanghai University, Shanghai 200444, China

[4]CAS Key Laboratory of Mechanical Behavior and Design of Materials, University of Science and Technology of China, Hefei, Anhui 230026, China

[5]Key Laboratory of Precision and Intelligent Chemistry, University of Science and Technology of China, Hefei, Anhui 230026, China

[6]Clean Nano Energy Center, State Key Laboratory of Metastable Materials Science and Technology, Yanshan University, Qinhuangdao, Hebei 066004, China

[7]ZepTools Technology Co., Ltd., Tongling, Anhui 244000, China

[8]Key Laboratory of Artificial Structures and Quantum Control, Shanghai Jiao Tong University, Shanghai 200240, China

[9]China Spallation Neutron Source, Institute of High Energy Physics, Chinese Academy of Sciences, Beijing 100049, China

[10]Instruments Center for Physical Science, University of Science and Technology of China, Hefei, Anhui 230026, China

[11]Shenzhen Key Laboratory of Advanced Energy Storage, Southern University of Science and Technology, Shenzhen, Guangdong 518055, China

[12]i-Lab, Suzhou Institute of Nano-Tech and Nano-Bionics, Chinese Academy of Sciences, Suzhou, Jiangsu 215123, China

[13]Shanghai Electrochemical Energy Device Research Center (SEED), Shanghai Jiao Tong University, Shanghai 200240, China

[14]Frontiers Science Center for Transformative Molecules, Shanghai Jiao Tong University, Shanghai 200240, China

[15]Future Battery Research Center, Global Institute of Future Technology, Shanghai Jiao Tong University, Shanghai 200240, China

[16]National Synchrotron Radiation Laboratory, Hefei, Anhui 230026, China

[†]These authors contributed equally to this work

*Corresponding authors: mach16@ustc.edu.cn (C.M.), sqshi@shu.edu.cn (S.S.)

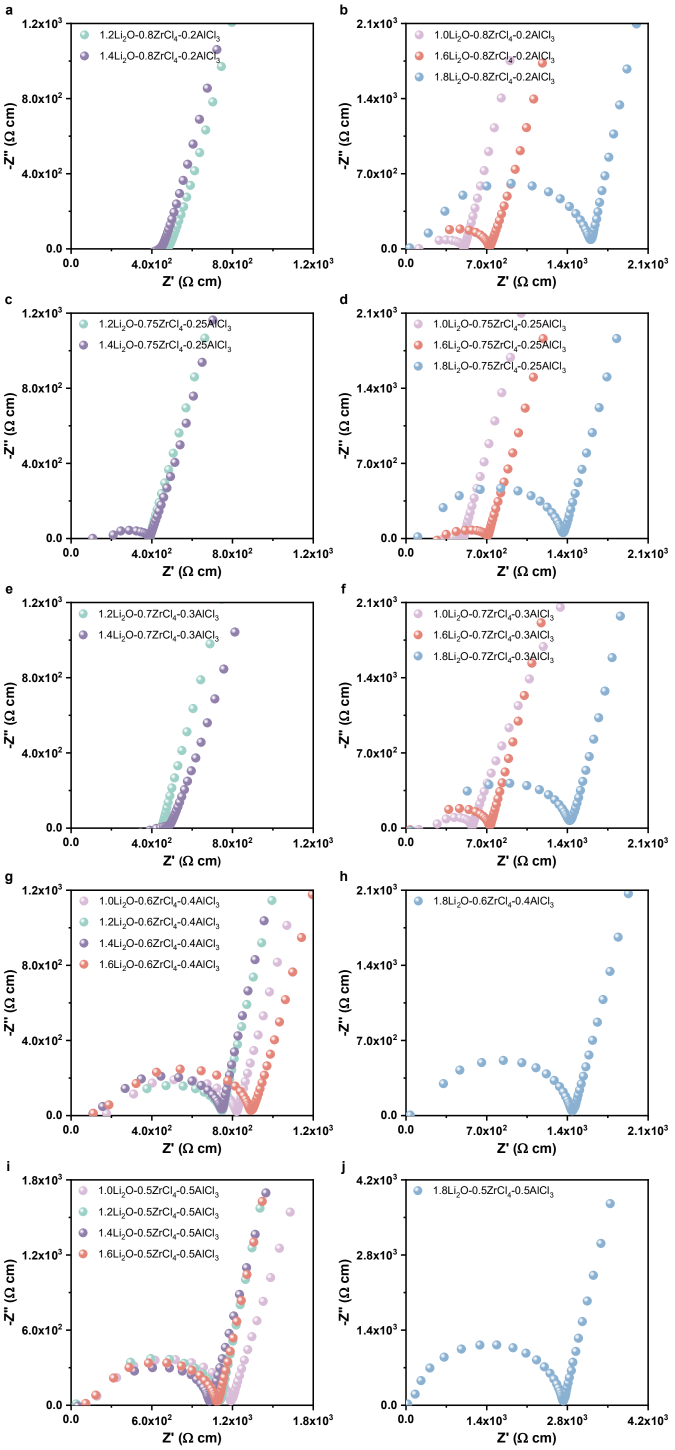

Supplementary Fig. 1 | Nyquist plots of xLi$_2$O-(1-y)ZrCl$_4$-yAlCl$_3$ at 25 °C. a Nyquist plots of xLi$_2$O-0.8ZrCl$_4$-0.2AlCl$_3$ with x = 1.2 and 1.4. **b** Nyquist plots of xLi$_2$O-0.8ZrCl$_4$-0.2AlCl$_3$ with x = 1.0, 1.6, and 1.8. **c** Nyquist plots of xLi$_2$O-0.75ZrCl$_4$-0.25AlCl$_3$ with x = 1.2 and 1.4. **d** Nyquist plots of xLi$_2$O-0.75ZrCl$_4$-0.25AlCl$_3$ with x = 1.0, 1.6, and 1.8. **e** Nyquist plots of xLi$_2$O-0.7ZrCl$_4$-0.3AlCl$_3$ with x = 1.2 and 1.4. **f** Nyquist plots of xLi$_2$O-0.7ZrCl$_4$-0.3AlCl$_3$ with x = 1.0, 1.6, and 1.8. **g** Nyquist plots of xLi$_2$O-0.6ZrCl$_4$-0.4AlCl$_3$ with x = 1.0, 1.2, 1.4, and 1.6. **h** Nyquist plot of 1.8Li$_2$O-0.6ZrCl$_4$-0.4AlCl$_3$. **i** Nyquist plots of xLi$_2$O-0.5ZrCl$_4$-0.5AlCl$_3$ with x = 1.0, 1.2, 1.4, and 1.6. **j** Nyquist plot of 1.8Li$_2$O-0.5ZrCl$_4$-0.5AlCl$_3$.

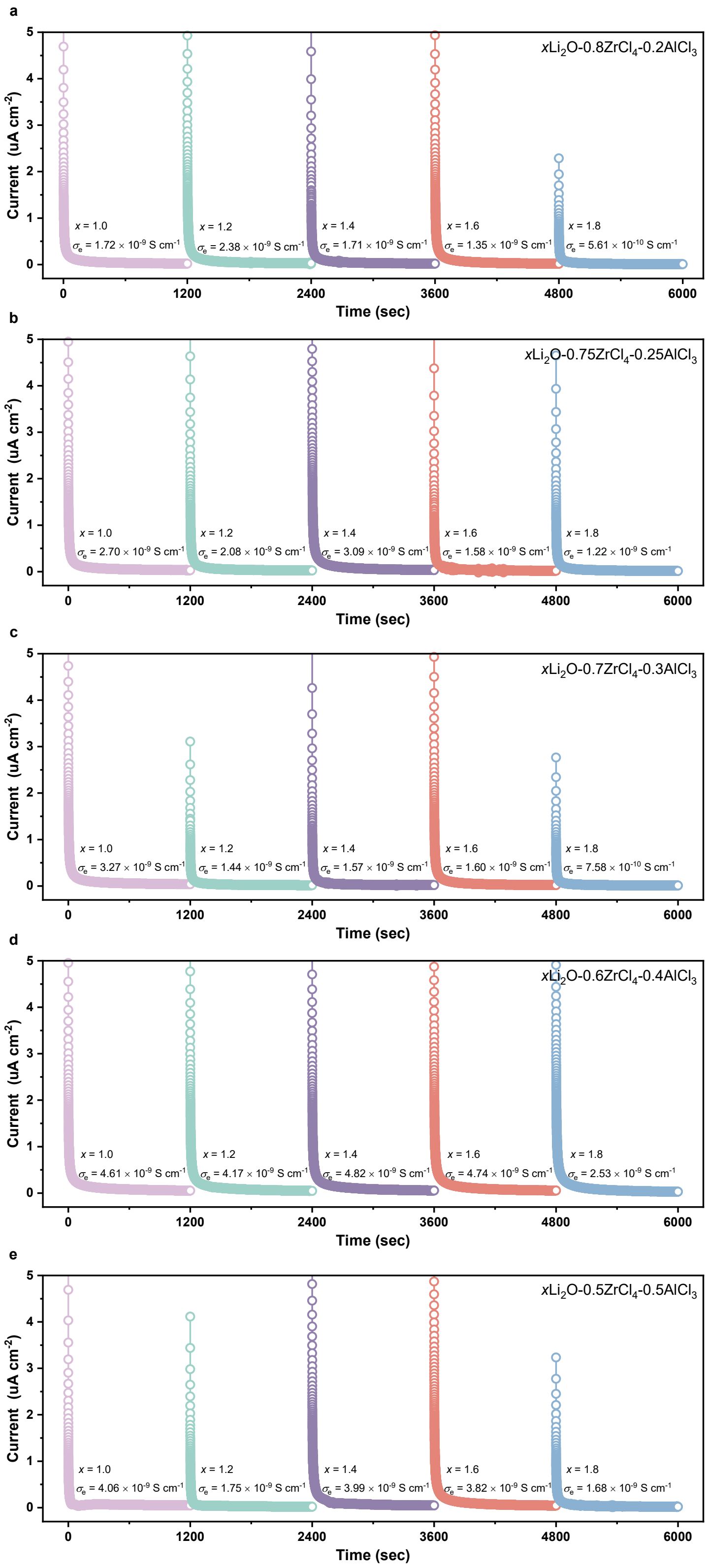

Supplementary Fig. 2 | Electronic conductivities of xLi$_2$O-(1-y)ZrCl$_4$-yAlCl$_3$. a–e Transient current behavior under an applied direct-current bias of 1.0 V with stainless steel electrodes at 25 °C for xLi$_2$O-(1-y)ZrCl$_4$-yAlCl$_3$ with y = 0.2 (**a**), 0.25 (**b**), 0.3 (**c**), 0.4 (**d**), and 0.5 (**e**).

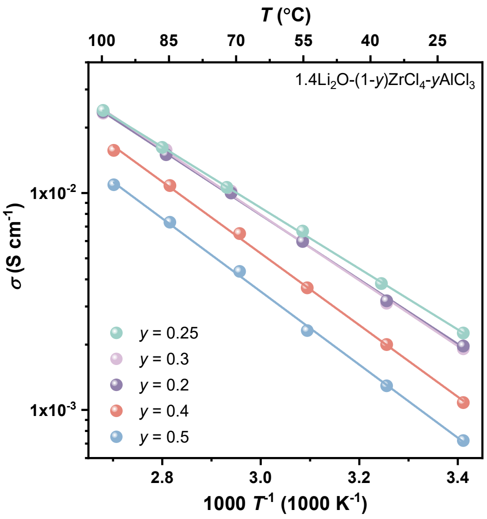

Supplementary Fig. 3 | Temperature-dependent ionic conductivities of 1.4Li_2O-(1-*y*)$ZrCl_4$-*y*$AlCl_3$. Arrhenius plots of the mechanochemically synthesized 1.4Li_2O-(1-*y*)$ZrCl_4$-*y*$AlCl_3$ with different *y*.

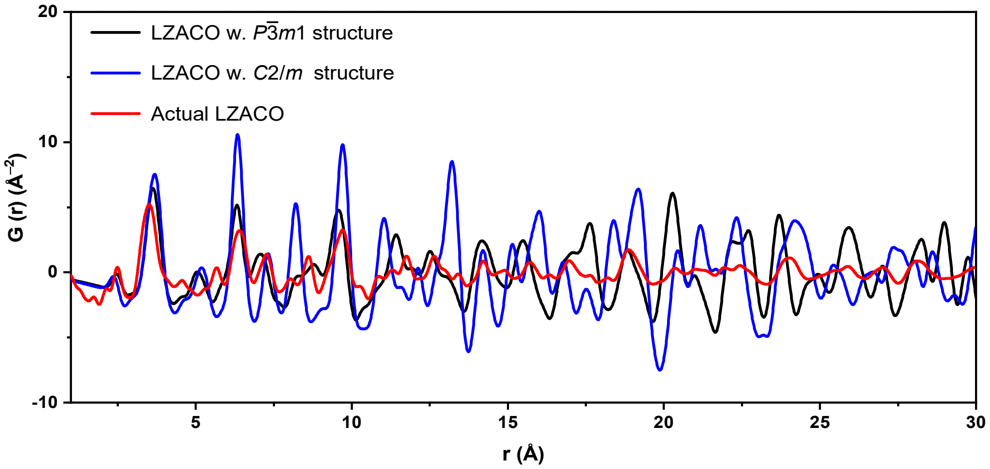

Supplementary Fig. 4 | PDF analysis of LZACO. PDF generated from the experimentally collected neutron total scattering data of LZACO, along with the simulated PDFs of the LZACO with the $P\bar{3}m1$ structure and that with the *C2/m* structure.

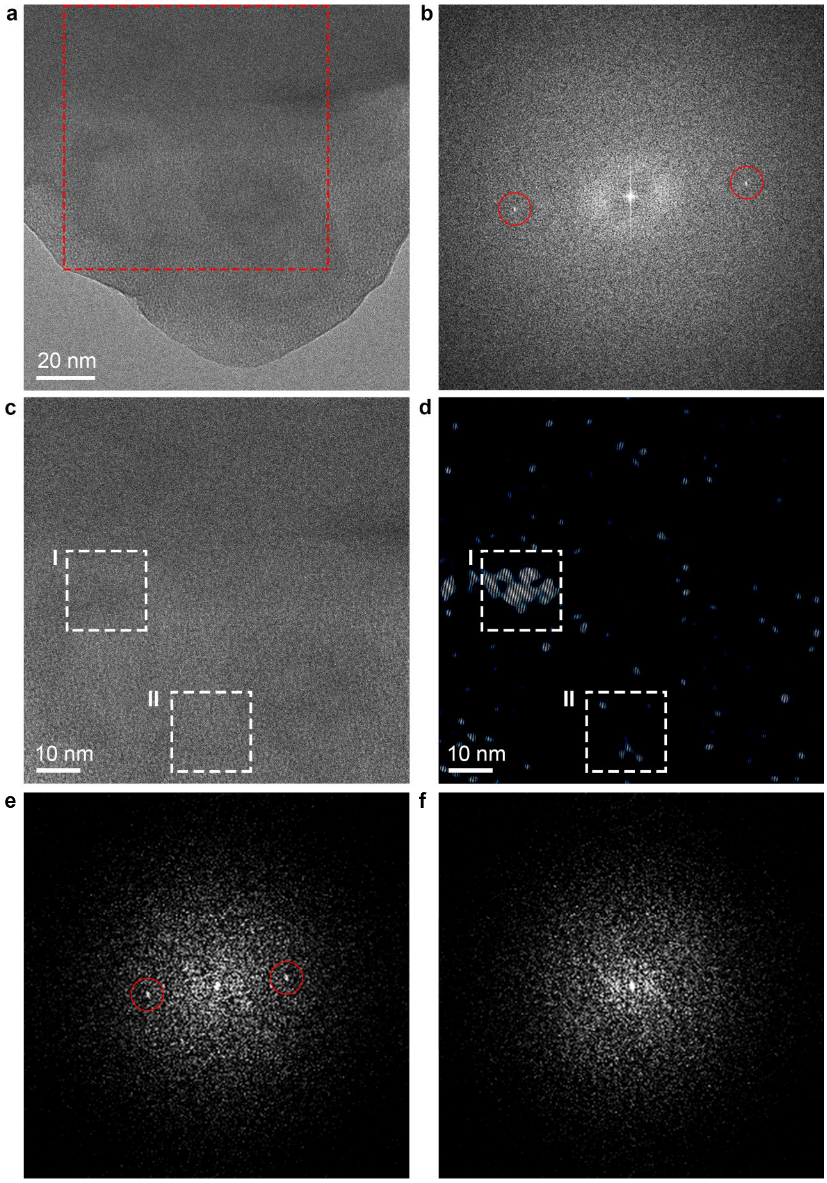

Supplementary Fig. 5 | TEM observation of LZACO. a HRTEM image of LZACO. **b** FFT pattern of the area delineated by the red dashed rectangle in (**a**). The spots arising from the crystalline phase are highlighted by the red circles. **c** HRTEM image of the area delineated by the red dashed rectangle in (**a**). **d** Inverse FFT pattern using the spots circled in red in (**b**). Region I in (**c**) are the same area as Region I in (**d**), while the same applies to Region II as well. **e, f** FFT patterns of Region I (**e**) and Region II (**f**) in (**c**), respectively, with the spots arising from the crystalline phase highlighted by the red circles.

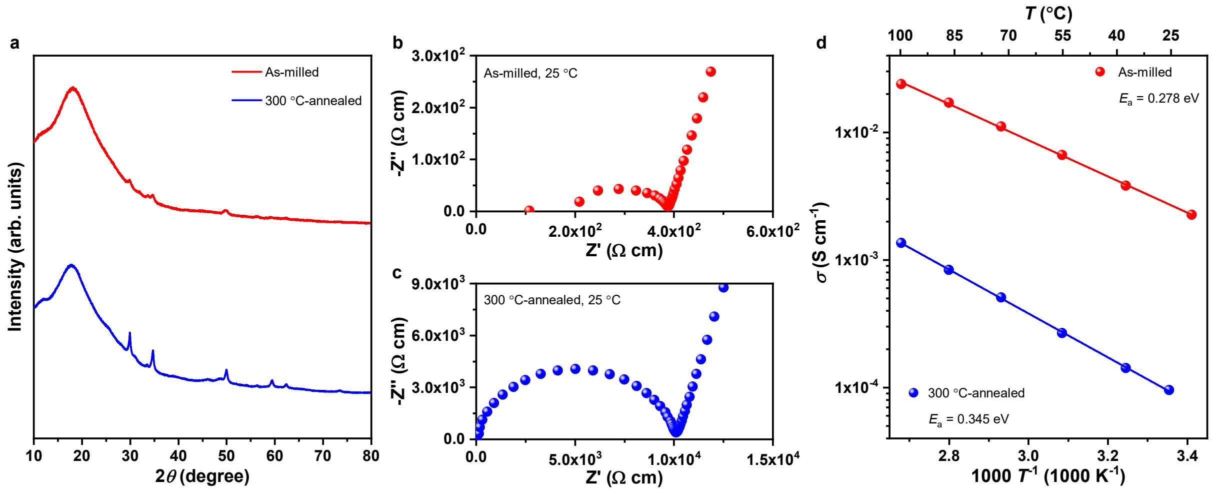

Supplementary Fig. 6 | Structure and ionic conductivity change of LZACO after 300 °C annealing. **a** XRD patterns of the as-milled and 300 °C-annealed LZACO. **b, c** The Nyquist plots of the as-milled (**b**) and 300 °C-annealed (**c**) LZACO at 25 °C. **d** Arrhenius plots of the as-milled and 300 °C-annealed LZACO.

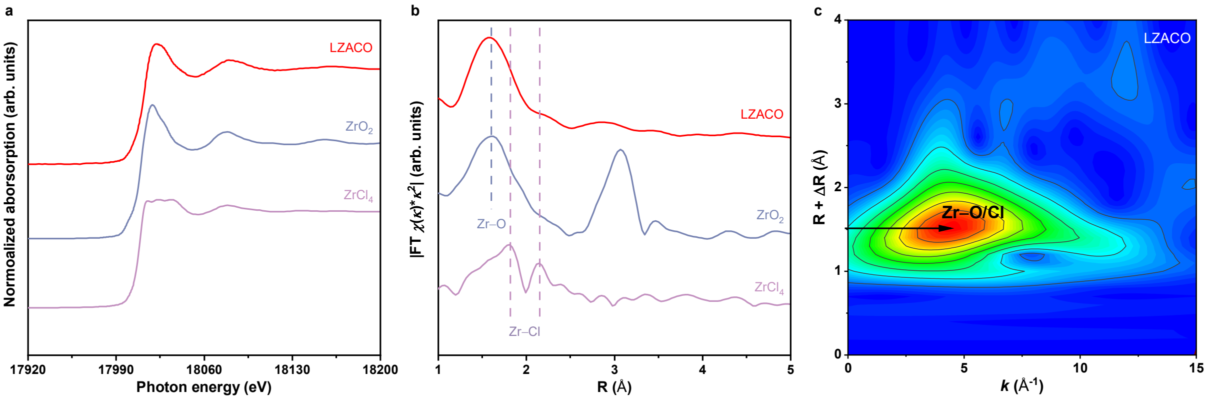

Supplementary Fig. 7 | XAS analyses for LZACO. a,b XANES (**a**) and FT-EXAFS (**b**) spectra of LZACO at the Zr *K*-edge. For comparison, the spectra of $ZrCl_4$ and ZrO_2 are also presented. **c** WT-EXAFS contour plot of LZACO at the Zr *K*-edge.

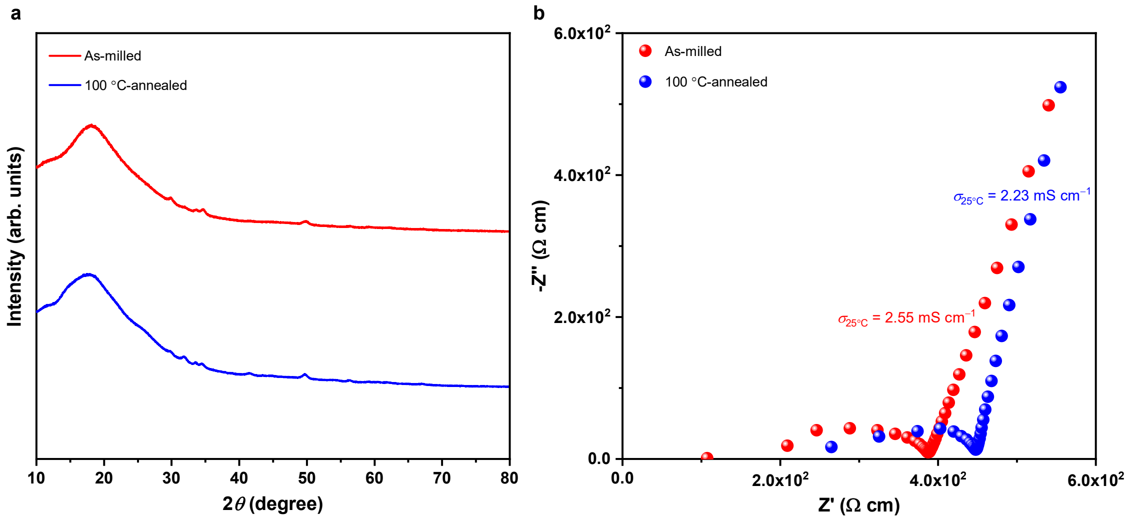

Supplementary Fig. 8 | Structure and ionic conductivity change of LZACO after 100 °C annealing. **a** XRD patterns of the as-milled LZACO and that annealed at 100 °C for 5 hours. **b** Nyquist plots of the as-milled LZACO and that annealed at 100 °C for 5 hours. The measurement was conducted at 25 °C.

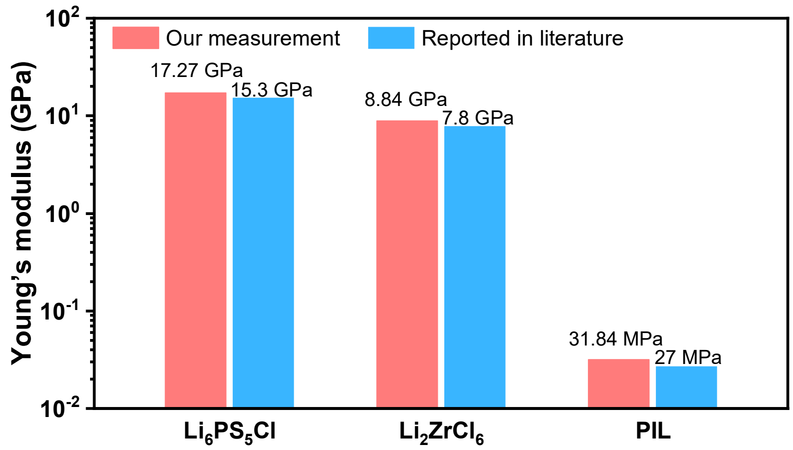

Supplementary Fig. 9 | Comparison of the Young's moduli we measured and those reported in literature. PIL represents a polymer solid electrolyte whose Young's modulus has been reported before in *Nat. Commun.* 14, 2301, 2023; it is prepared via ultraviolet light-initiated copolymerization of 1-allyl-1-methyl-pyrrolidinium bis(trifluoromethanesulfonyl) imide ionic liquid, vinyl ethylene carbonate, and polyfluorinated crosslinker 2,2,3,3,4,4,5,5-octafluoro-1,6-hexanediol diacrylate (OFHDODA) blended with lithium salt (LiTFSI) in the molar ratio of 8:3:2. The reference data for Li_6PS_5Cl and Li_2ZrCl_6 are taken from *Joule* 8, 1–16, 2024 and *Angew. Chem. Int. Ed.* 64 (36), e202510359, 2025, respectively.

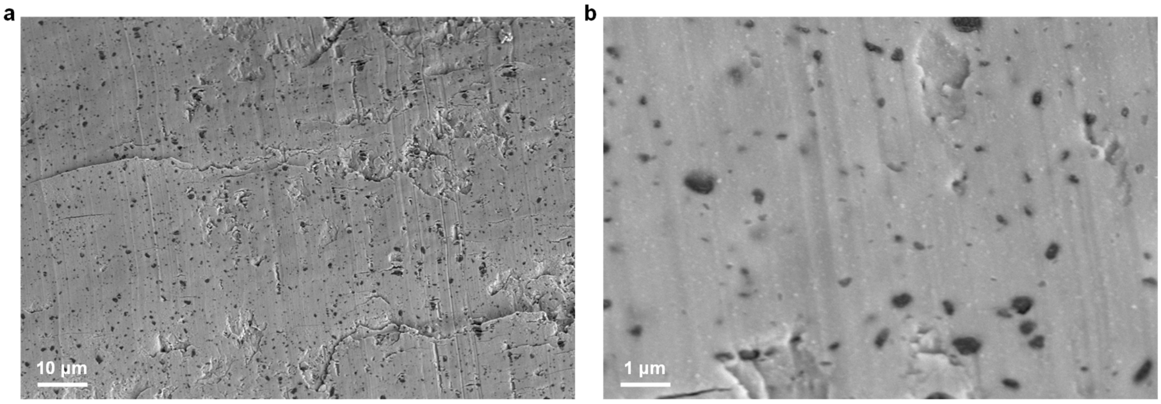

Supplementary Fig. 10 | SEM observation of the cold-pressed LZACO pellet. a SEM image of the surface of the cold-pressed LZACO pellet fabricated under 300 MPa (the same as that for the cold-pressed pellet used in the nanoindentation experiments). **b** SEM image of the same pellet at a higher magnification.

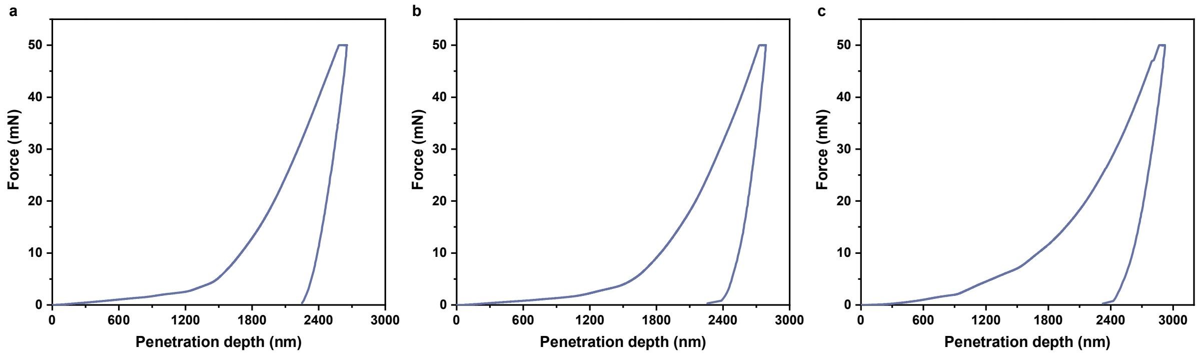

Supplementary Fig. 11 | Representative load-displacement curves from nanoindentation tests. a–c Representative nanoindentation load-displacement curves for the cold-pressed LZACO pellet fabricated under 300 MPa.

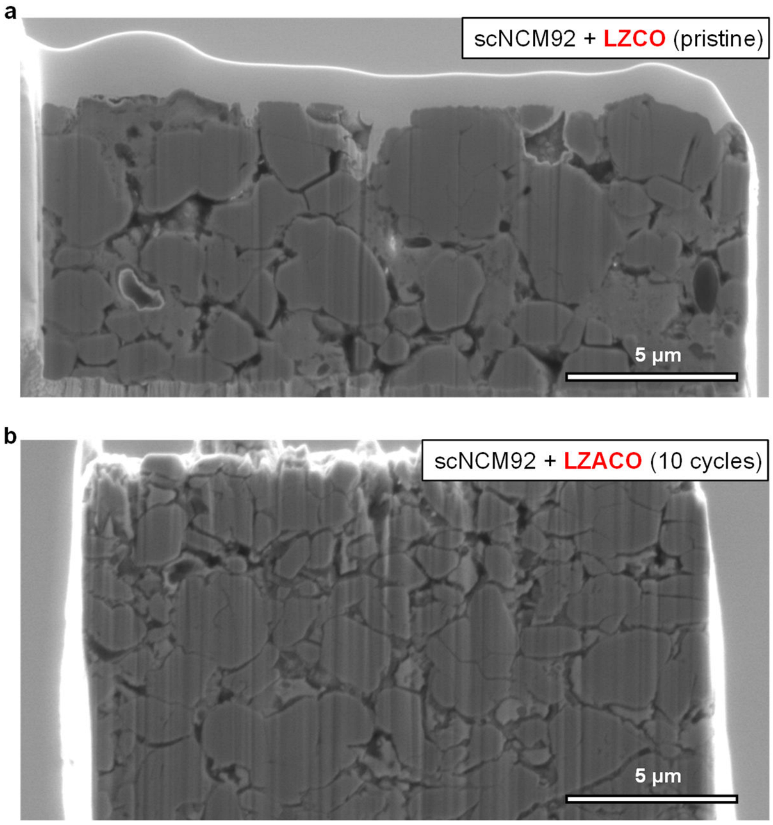

Supplementary Fig. 12 | Morphologies of the composite positive electrodes based on different solid electrolytes. a SEM image of the composite positive electrode of the Li-In | LPSCl-LZCO | scNCM92 cell prior to cycling. **b** SEM image of the composite positive electrode of the Li-In | LPSCl-LZACO | scNCM92 cell after 10 cycles under the conditions shown in Fig. 2d (20 mA g^{-1}, 2.8–4.3 V vs. Li/Li^{+}, 25 °C, 190 MPa).

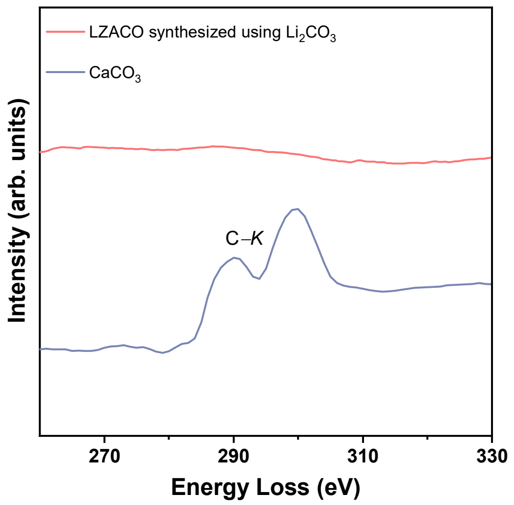

Supplementary Fig. 13 | Evidence for the complete decopmosition of L_2CO_3 during the synthesis of LZACO. Electron energy-loss spectra of the LZACO synthesized using Li_2CO_3, $ZrCl_4$, and $AlCl_3$ as the raw materials after 4-h planetary mill. For comparison, the C-*K* spectrum of $CaCO_3$ is also displayed.

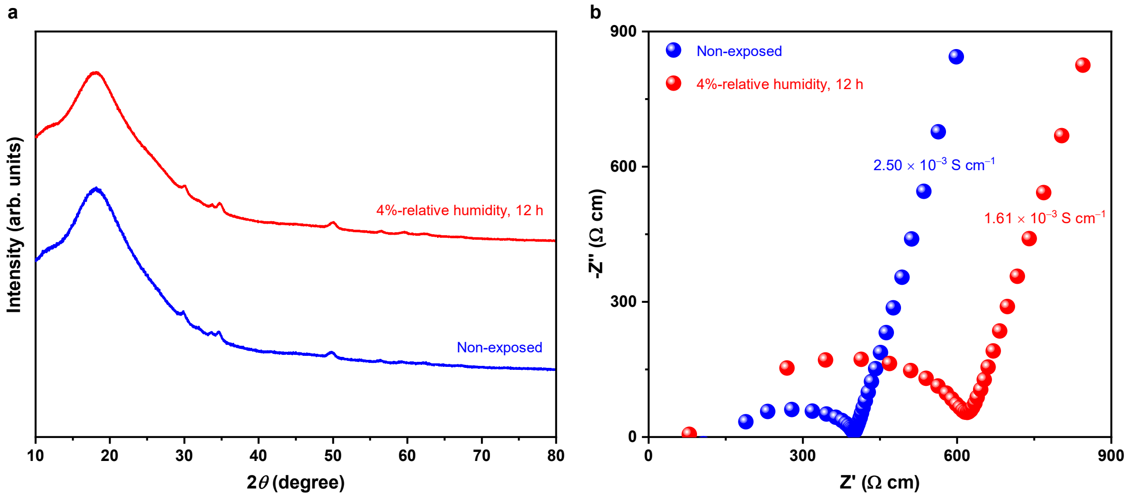

Supplementary Fig. 14 | Humidity tolerance test conducted in an environmental chamber. a XRD patterns of the LZACO powders before and after exposure to the air with 4% relative humidity at 25 °C. **b** Nyquist plots of LZACO before and after exposure to the air with 4% relative humidity at 25 °C. The exposure experiment was conducted using the powder samples, while the EIS measurements were conducted on the pellets prepared by cold pressing the exposed or non-exposed powders under 300 MPa, at 25 °C.

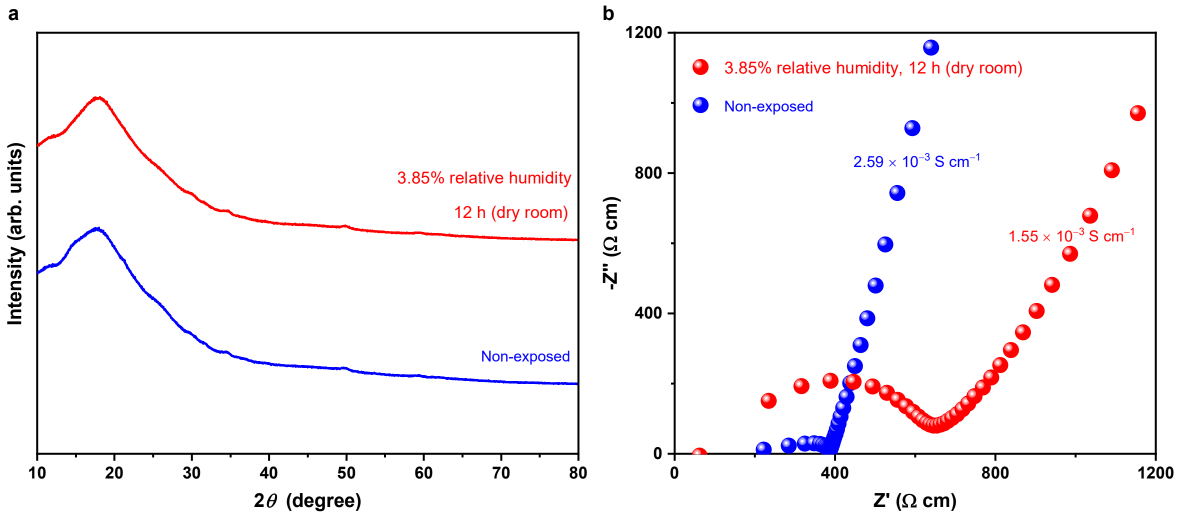

Supplementary Fig. 15 | Humidity tolerance test conducted in a dry room. a XRD patterns of the LZACO powders before and after exposure to the air with 3.85% relative humidity at 25 °C. **b** Nyquist plots of LZACO before and after exposure to the air with 3.85% relative humidity at 25 °C. The exposure experiment was conducted using the powder samples, while the EIS measurements were conducted on the pellets prepared by cold pressing the exposed or non-exposed powders under 300 MPa, at 25 °C.

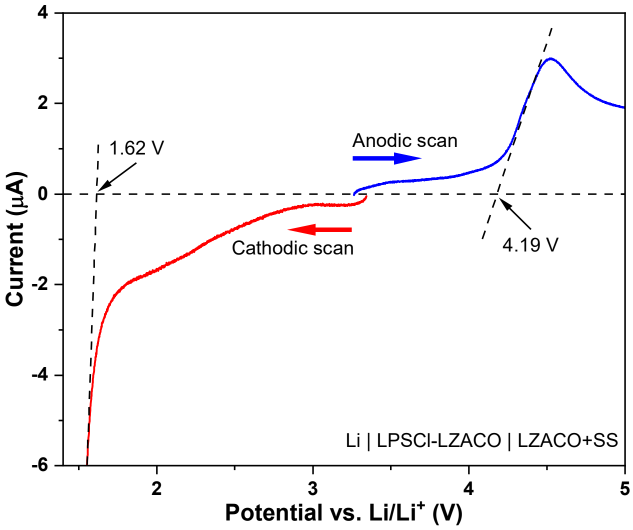

Supplementary Fig. 16 | Electrochemical stability window of LZACO. LSV curves of the Li | LPSCl-LZACO | LZACO + SS cell at 0.1 mV s^{-1}. The measurements were conducted at 25 °C.

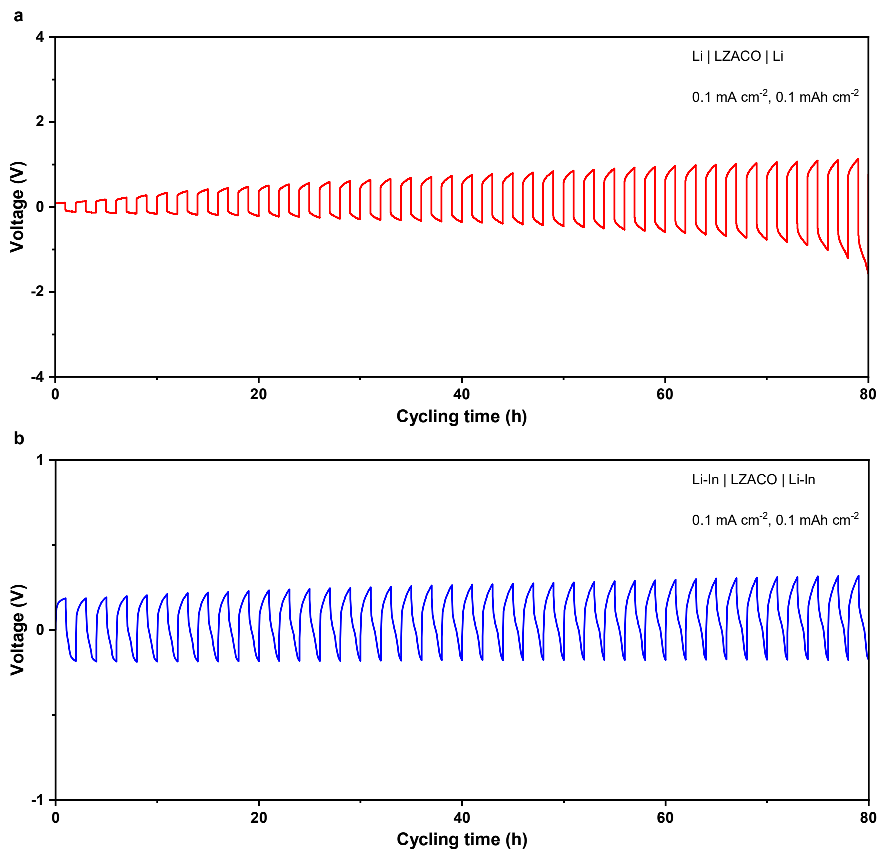

Supplementary Fig. 17 | Compatibility between LZACO and different anodes. a,**b** Galvanostatic cycling profiles of the symmetric Li | LZACO | Li cell (**a**) and the symmetric Li-In | LZACO | Li-In cell (**b**) at 0.1 mA cm^{-2} (1 hour per cycle) and 25 °C.

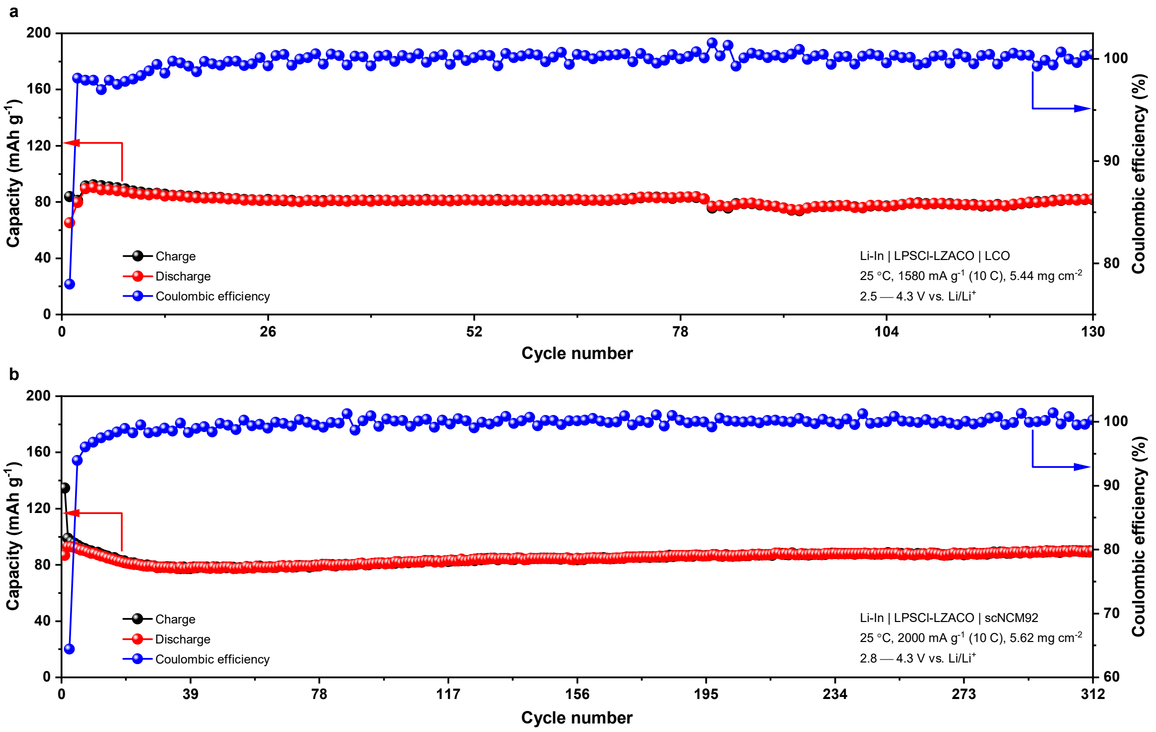

Supplementary Fig. 18 | Formation cycles prior to the long-term cycling. a Formation cycles prior to the long-term cycling of the Li-In | LPSCl-LZACO | LCO cell shown in Fig. 4g of the main text. **b** Formation cycles prior to the long-term cycling of the Li-In | LPSCl-LZACO | scNCM92 cell shown in Fig. 4h of the main text.

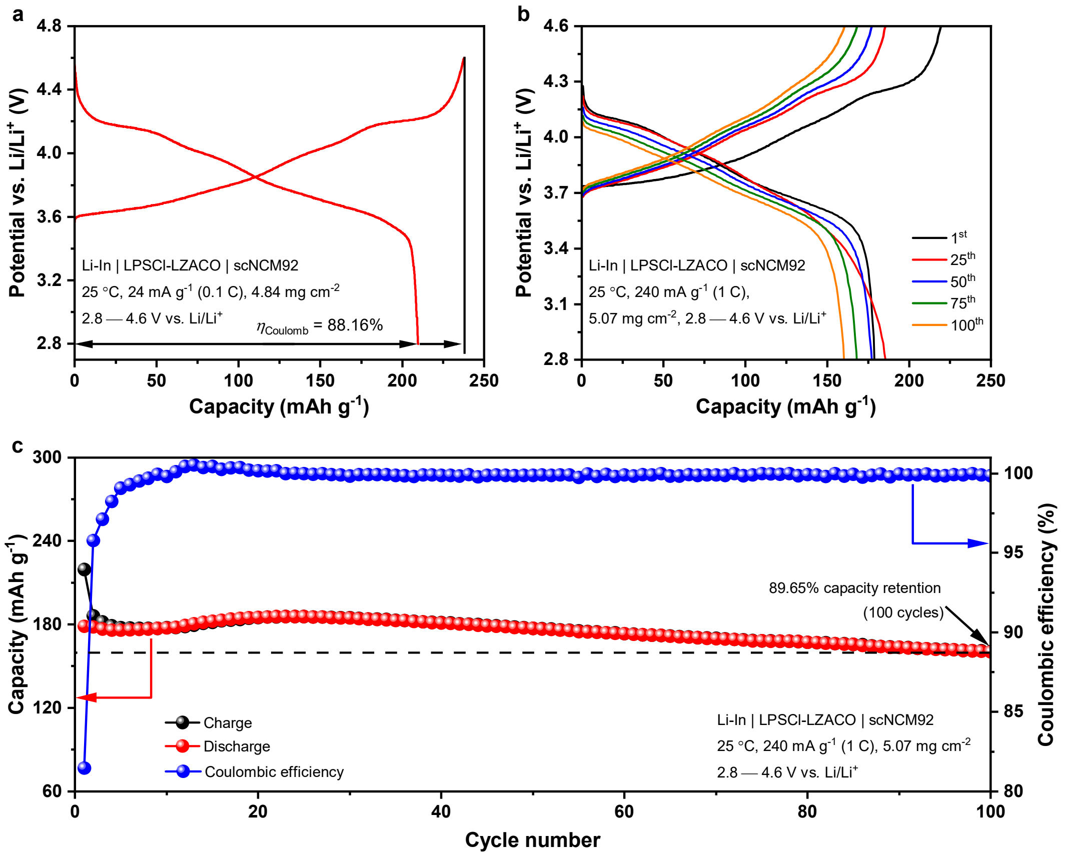

Supplementary Fig. 19 | Cycling performance of the LZACO-based cell with the 4.6 V vs. Li/Li$^+$ upper cutoff potential. a Initial charge/discharge profiles of the Li-In | LPSCl-LZACO | scNCM92 cell between 2.8 and 4.6 V vs. Li/Li$^+$ under 24 mA g^{-1} (0.1 C) at 25 °C. η_{Coulomb} represents the Coulombic efficiency. **b** Charge/discharge profiles of the Li-In | LPSCl-LZACO | scNCM92 cell at different cycles under 240 mA g^{-1} (1 C) and 25 °C. **c** Long-term cycling performance of the Li-In | LPSCl-LZACO | scNCM92 cell under 240 mA g^{-1} (1 C) at 25 °C. The potential vs. Li/Li$^+$ is calculated by adding the cell voltage and the potential of the Li-In alloy anode, i.e., 0.62 vs. Li/Li$^+$. The stacking pressure during cycling is 190 MPa.

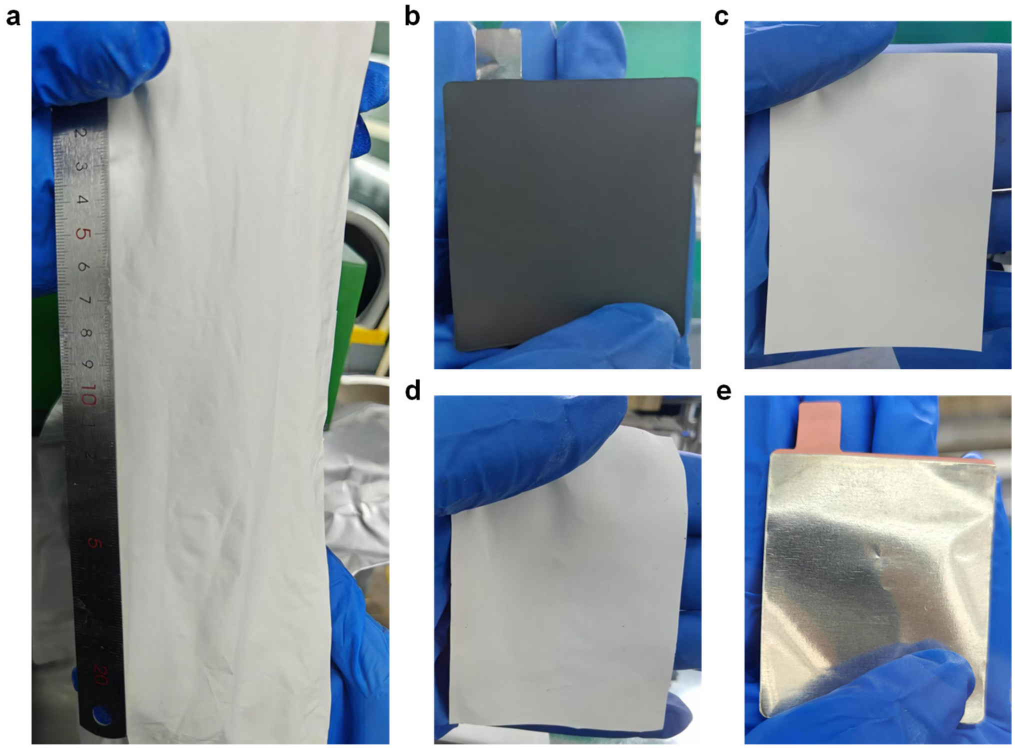

Supplementary Fig. 20 | Films and foils used for pouch-cell fabrication. a LZACO film prepared by the dry-film technology. **b**–**e** Composite-positive-electrode film (**b**), LZACO film (**c**), LPSCl film (**d**), and Li metal negative electrode (**e**) for all-solid-state pouch cells. The composite-positive-electrode film, LZACO film, and LPSCl film are all prepared by the dry-film technology.

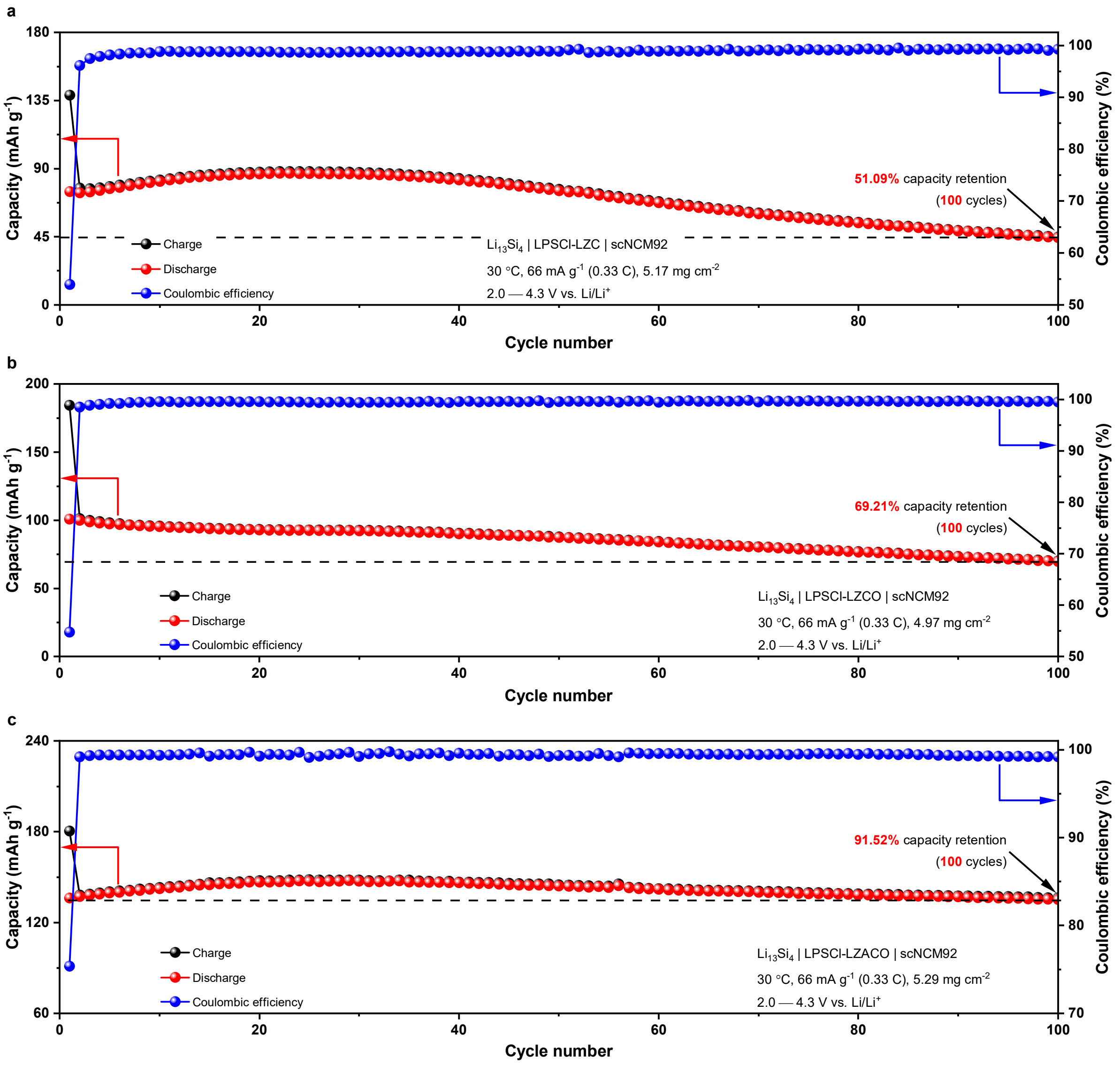

Supplementary Fig. 21 | Low-pressure cell performance enabled by different Zr-based (oxy)chloride solid electrolytes. a–c Long-term cycling performance of the $Li_{13}Si_4$ | LPSCl-LZC | scNCM92 cell (**a**), $Li_{13}Si_4$ | LPSCl-LZCO | scNCM92 cell (**b**), and $Li_{13}Si_4$ | LPSCl-LZACO | scNCM92 cell (**c**) under 5 MPa at 30 °C.

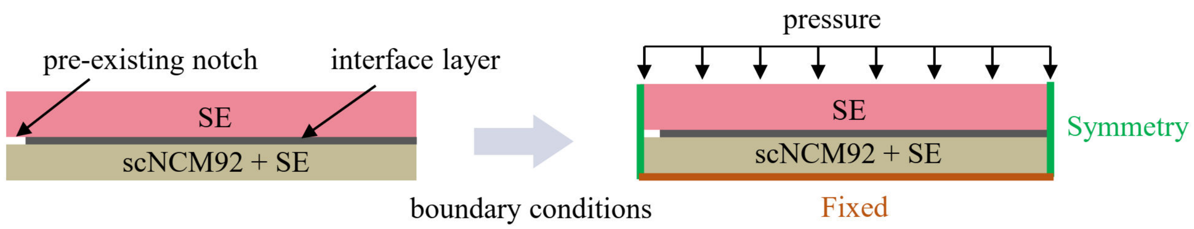

Supplementary Fig. 22 | Schematic illustration of the model used for the phase-field modeling. The abbervation “SE” represents “solid electrolyte”.

Supplementary Table 1. Instrument depreciation costs for producing different solid electrolytes. The Li_2ZrCl_6 density used for the cost analysis is 2.56 g cm^{-3}, as previously reported in *Nat. Commun.* 12, 4410, 2021, while the LZACO density is measured to be 2.15 g cm^{-3} (details in Methods). "SE" represents "solid electrolyte".

		Li_2ZrCl_6	LZACO (from Li_2O)	LZACO (from Li_2CO_3)
Amount of SE produced per batch (kg)		28		
Planetary mill	Cost per instrument ($)	15169.46		
	No. of instruments involved	28		
	Depreciation period (year)	7		
	Effective milling time (h)	45	30	4
	Instrument working time (h)	90	60	8
	Depreciation cost of this instrument ($ L^{-1})	57.02	31.91	4.25
Walk-in enclosure	Cost per instrument ($)	164384.56		
	No. of instruments involved	1		
	Depreciation period (year)	15		
	Instrument working time (h)	92	62	10
	Depreciation cost of this instrument ($ L^{-1})	10.53	5.96	0.96
Depreciation cost of all the instruments ($ L^{-1})		67.55	37.87	5.21

Supplementary Table 2. Electricity costs for producing different solid electrolytes. The Li_2ZrCl_6 density used for the cost analysis is 2.56 g cm^{-3}, as previously reported in *Nat. Commun.* 12, 4410, 2021, while the LZACO density is measured to be 2.15 g cm^{-3} (details in Methods). "SE" represents "solid electrolyte".

		Li_2ZrCl_6	LZACO (from Li_2O)	LZACO (from Li_2CO_3)
Amount of SE produced per batch (kg)		28		
Electricity price (\$ kWh^{-1})		0.084		
Planetary mill	Power (kW)	3.0		
	No. of instruments involved	28		
	Effective milling time (h)	45	30	4
	Electricity cost of this instrument (\$ L^{-1})	29.04	16.25	2.17
Walk-in enclosure	No. of instruments involved	1		
	Relative humidity	4%		
	Power without people inside (kW)	0.31		
	Power with people inside (kW)	2.51		
	Time without people inside (h)	90	60	8
	Time with people inside (h)	2	2	2
	Electricity cost of this instrument (\$ L^{-1})	0.25	0.15	0.05
Electricity cost of all the instruments (\$ L^{-1})		29.29	16.40	2.22

Supplementary Table 3. Personnel costs for producing different solid electrolytes. The Li_2ZrCl_6 density used for the cost analysis is 2.56 g cm^{-3}, as previously reported in *Nat. Commun.* 12, 4410, 2021, while the LZACO density is measured to be 2.15 g cm^{-3} (details in Methods). "SE" represents "solid electrolyte".

		Li_2ZrCl_6	LZACO (from Li_2O)	LZACO (from Li_2CO_3)
Amount of SE produced per batch (kg)		28		
Labor cost person (\$ h^{-1})		7.04		
Planetary mill	No. of instrument involved	28		
	No. of instrument each person is in charge of	4		
	No. of people needed	7		
	Time each person needs to spend (h)	2		
	Personnel cost for this instrument (\$ L^{-1})	9.01	7.57	7.57

Supplementary Table 4. Plant area costs for producing different solid electrolytes. The Li_2ZrCl_6 density used for the cost analysis is 2.56 g cm^{-3}, as previously reported in *Nat. Commun.* 12, 4410, 2021, while the LZACO density is measured to be 2.15 g cm^{-3} (details in Methods). "SE" represents "solid electrolyte".

	Li_2ZrCl_6	LZACO (from Li_2O)	LZACO (from Li_2CO_3)
Amount of SE produced per batch (kg)	28		
Industrial facility rent per month ($ m^{-2})	4.92		
Area of the walk-in enclosure for producing the SEs (m^2)	60		
Time needed for each batch of production (h)	92	62	10
Plant area cost ($ L^{-1})	3.45	1.95	0.31

Supplementary Table 5. Raw materials cost, processing cost, and production cost for different solid electrolytes. The Li_2ZrCl_6 density used for the cost analysis is 2.56 g cm^{-3}, as previously reported in *Nat. Commun.* 12, 4410, 2021, while the LZACO density is measured to be 2.15 g cm^{-3} (details in Methods).

Solid electrolyte	Raw materials cost ($ L^{-1})	Processing cost ($ L^{-1})	Production cost ($ L^{-1})
Li_2ZrCl_6	30.71	109.30	140.01
LZACO (from Li_2O)	92.97	63.79	156.76
LZACO (from Li_2CO_3)	28.39	15.31	43.70

Supplementary Table 6. Average market prices of the industrial commodity chemicals in 2024 and the sources from which these prices are acquired.

Commodity chemical	Average market price in 2024 ($ kg^{-1})	Sources
Li_2O	210.67	LB Group Co., Ltd.<br>Shandong Hongyang Chemical Co., Ltd.<br>Chengxin Lithium Group Co., Ltd.<br>CBC Metal (https://www.cbcie.com/)<br>Asian Metal (https://www.asianmetal.cn/)<br>Shanghai Metals Market (https://www.smm.cn/)
LiCl	14.04	
Li_2CO_3	12.64	
$ZrCl_4$	11.24	
$AlCl_3$	0.84	

Supplementary Table 7. Comparison of the initial discharge capacity and Coulombic efficiency between the LZACO-based cell in Fig. 4a and those with the similar cell configuration.

PEAM	Catholyte	Specific current (mA g^{-1})	Initial discharge capacity (mAh g^{-1})	Coulombic efficiency	Ref.
LCO	LZACO	31.6 (0.2 C)	152.6	97.26%	This work
LCO	$Li_{1.75}ZrCl_{4.75}O_{0.5}$	79 (0.5 C)	145.6	97.73%	(3)
LCO	$Li_{3.4}ZrCl_{4.6}O_{1.4}$	14 (0.1 C)	160.9	97.3%	(45)
LCO	$Li_{5/3}Cr_{1/3}Zr_{1/3}Cl_4$	~31.6 (0.2 C)	108.2	96.09%	(71)
LCO	Li_2ZrCl_6	14 (0.1 C)	137	97.9%	(47)
LCO	$Li_3ZrCl_4O_{1.5}$	70 (0.5 C)	~113.5	94.7%	(25)
LCO	$LiNbOCl_4$	~14 (0.1 C)	~125	95%	(21)
LCO	Li_3ScCl_6	~14.6 (0.1 C)	126.2	90.3%	(50)

Supplementary Table 8. Comparison of the initial discharge capacity and Coulombic efficiency between the LZACO-based cell in Fig. 4d and those with the similar cell configuration.

PEAM	Catholyte	Specific current ($mA\ g^{-1}$)	Initial discharge capacity ($mAh\ g^{-1}$)	Coulombic efficiency	Ref.
$LiNi_{0.92}Co_{0.06}Mn_{0.02}O_2$	LZACO	20 (0.1 C)	180.8	90.27%	This work
$LiNi_{0.8}Co_{0.1}Mn_{0.1}O_2$	$Li_{1.75}ZrCl_{4.75}O_{0.5}$	20 (0.1 C)	173.96	87.31%	(3)
$LiNi_{0.88}Co_{0.11}Al_{0.01}O_2$	Li_2ZrCl_6	~24 (0.1 C)	206	85.8%	(49)
$LiNi_{0.5}Co_{0.2}Mn_{0.3}O_2$	$Li_3Zr_2Si_2PO_{12}$	15 (0.1 C)	162	85.8%	(75)
$LiNi_{0.5}Co_{0.2}Mn_{0.3}O_2$	$Li_3PW_{12}O_{40}$	~15 (0.1 C)	165.6	98.1%	(57)

Supplementary Table 9. Cycling performance of the LZACO-based ASSLBs and those based on other solid electrolytes when the PEAM is LCO with the ordinary mass loading (5–10 mg cm^{-2}).

PEAM	Catholyte	Specific current (mA g^{-1})	Specific capacity (mAh g^{-1})	Capacity retention	Ref.	Cost-effective catholyte
LCO	LZACO	1580 (10 C)	84.8 (417 cycles)	98% (417 cycles)	This work	Yes
			82.1 (746 cycles)	95% (746 cycles)		
			75.3 (1577 cycles)	90% (1577 cycles)		
			70.9 (2059 cycles)	82% (2059 cycles)		
LCO	$Li_{1.75}ZrCl_{4.75}O_{0.5}$	700 (5 C)	102 (150 cycles)	92.4% (150 cycles)	(3)	
LCO	$Li_{3.4}ZrCl_{4.6}O_{1.4}$	140.7 (1 C)	122 (600 cycles)	94% (600 cycles)	(45)	
LCO	$Li_{5/3}Cr_{1/3}Zr_{1/3}Cl_4$	158 (1 C)	79.1 (410 cycles)	86% (410 cycles)	(71)	
LCO	Li_2ZrCl_6	82 (0.5 C)	139.4 (100 cycles)	90.5% (100 cycles)	(49)	
LCO	ZrO_2-$2Li_2ZrCl_5F$	82 (0.5 C)	134.5 (100 cycles)	92.1% (100 cycles)	(16)	
LCO	$Li_3ZrCl_4O_{1.5}$	70 (0.5 C)	106 (240 cycles)	90% (240 cycles)	(25)	
LCO	$LiNbOCl_4$	42 (0.3 C)	122 (100 cycles)	97% (100 cycles)	(21)	No
LCO	Li_3ScCl_6	14.6 (0.1 C)	104.5 (160 cycles)	82.8% (160 cycles)	(50)	
LCO	$Li_3YBr_3Cl_3$	14 (0.1 C)	108 (58 cycles)	87.1% (58 cycles)	(18)	

Supplementary Table 10. Cycling performance of the LZACO-based ASSLBs and those based on other solid electrolytes when the PEAM is Li(Ni,Co,Mn,Al)O_2 with the ordinary mass loading (5−10 mg cm^{-2}).

PEAM	Catholyte	Specific current (mA g^{-1})	Specific capacity (mAh g^{-1})	Capacity retention	Ref.	Cost-effective catholyte
$LiNi_{0.92}Co_{0.06}Mn_{0.02}O_2$	LZACO	2000 (10 C)	90 (697 cycles)	98% (697 cycles)	This work	Yes
			87 (1018 cycles)	95% (1018 cycles)		
			82.2 (2776 cycles)	90% (2776 cycles)		
			72.7 (4208 cycles)	80% (4208 cycles)		
$LiNi_{0.8}Co_{0.1}Mn_{0.1}O_2$	$Li_{1.75}ZrCl_{4.75}O_{0.5}$	1000 (5 C)	70.2 (2082 cycles)	70.34% (2082 cycles)	(3)	
$LiNi_{0.88}Co_{0.11}Mn_{0.01}O_2$	ZrO_2-$2Li_2ZrCl_5F$	400 (2 C)	114.8 (2000 cycles)	82% (2000 cycles)	(16)	
$LiNi_{0.83}Co_{0.11}Mn_{0.06}O_2$	$Li_3ZrCl_4O_{1.5}$	200 (1 C)	123.4 (300 cycles)	90.1% (300 cycles)	(25)	
$LiNi_{0.88}Co_{0.09}Mn_{0.03}O_2$	AlOCl-2LiCl	200 (1 C)	103.9 (1600 cycles)	84.9% (1600 cycles)	(41)	
$LiNi_{0.88}Co_{0.11}Al_{0.01}O_2$	Li_2ZrCl_6	~120 (0.5 C)	169 (100 cycles)	91.3% (100 cycles)	(49)	
$LiNi_{0.83}Co_{0.11}Mn_{0.06}O_2$	$SmCl_3 \cdot 0.5Li_2ZrCl_6$	100 (0.5 C)	115 (600 cycles)	85% (600 cycles)	(74)	
$LiNi_{0.5}Co_{0.2}Mn_{0.3}O_2$	$Li_3Zr_2Si_2PO_{12}$	15 (0.1 C)	146 (100 cycles)	90.1% (100 cycles)	(75)	
$LiNi_{0.5}Co_{0.2}Mn_{0.3}O_2$	$Li_3PW_{12}O_{40}$	15 (0.1 C)	151.5 (200 cycles)	91.5% (200 cycles)	(57)	
$LiNi_{0.88}Co_{0.09}Mn_{0.03}O_2$	$Li_{1.5}La_{0.2}Ce_{0.2}Zr_{0.2}Hf_{0.2}Ta_{0.2}Cl_{5.3}$	~800 (4 C)	88 (3000 cycles)	80% (3000 cycles)	(17)	No
$LiNi_{0.88}Co_{0.07}Mn_{0.05}O_2$	Li_2TaCl_7	600 (3 C)	70 (3000 cycles)	70% (3000 cycles)	(12)	
$LiNi_{0.85}Co_{0.1}Mn_{0.05}O_2$	$Li_2In_{1/3}Sc_{1/3}Cl_4$	541 (3 C)	71 (3000 cycles)	80% (3000 cycles)	(23)	
$LiNi_{0.83}Co_{0.11}Mn_{0.06}O_2$	$1.6Li_2O$-$TaCl_5$	400 (2 C)	96 (2400 cycles)	90.7% (2400 cycles)	(24)	

Supplementary Table 11. Cycling performance of the LZACO-based ASSLBs and those based on other solid electrolytes when the PEAM is LCO with the mass loading above 20 mg cm^{-2}.

PEAM	Catholyte	PEAM loading (mg cm^{-2})	Current density (mA cm^{-2})	Areal capacity (mAh cm^{-2})	Capacity retention	Ref.	Cost-effective catholyte
LCO	LZACO	28.17	0.445 (C/10)	3.63 (100 cycles)	85.78% (100 cycles)	This work	Yes
LCO	$Li_2In_{1/3}Sc_{1/3}Cl_4$	27.09	1.24 (C/3)	3 (180 cycles)	81.1% (180 cycles)	(23)	No
LCO	Li_3InCl_6	25	0.5 (C/10)	3.75 (150 cycles)	75% (150 cycles)	(28)	

Supplementary Table 12. Cycling performance of the LZACO-based ASSLBs and those based on other solid electrolytes when the PEAM is $Li(Ni,Co,Mn,Al)O_2$ with the mass loading above 20 mg cm^{-2}.

PEAM	Catholyte	PEAM loading (mg cm^{-2})	Current density (mA cm^{-2})	Areal capacity (mAh cm^{-2})	Capacity retention	Ref.	Cost-effective catholyte
$LiNi_{0.92}Co_{0.06}Mn_{0.02}O_2$	LZACO	25.75	0.515 (C/10)	3.92 (100 cycles)	90.11% (100 cycles)	This work	Yes
$LiNi_{0.92}Co_{0.05}Mn_{0.03}O_2$	Li_2TaCl_7	24.3	1.0 (0.19 C)	3.5 (110 cycles)	70% (110 cycles)	(77)	No
$LiNi_{0.88}Co_{0.07}Mn_{0.05}O_2$	Li_2TaCl_7	25.1	1.0 (C/5)	3.92 (75 cycles)	80% (75 cycles)	(12)	
$LiNi_{0.88}Co_{0.07}Mn_{0.05}O_2$	Li_2NbOCl_5	31.5	1.0 (C/5)	4.06 (95 cycles)	76% (95 cycles)	(12)	
$LiNi_{0.85}Co_{0.1}Mn_{0.05}O_2$	$Li_2In_{1/3}Sc_{1/3}Cl_4$	21.59	0.49 (C/8)	4.14 (80 cycles)	~100% (80 cycles)	(23)	
$LiNi_{0.83}Co_{0.11}Mn_{0.06}O_2$	$Li_{2.73}Ho_{1.09}Cl_6$	27.42	0.55 (C/10)	4.49 (100 cycles)	86.91% (100 cycles)	(78)	

Supplementary Table 13. Low-pressure cycling performance of the LZACO-based ASSLBs and those based on other solid electrolytes.

PEAM	Catholyte	Specific current (mA g^{-1})	Temperature (°C)	Stacking pressure (MPa)	Capacity retention	Ref.
$LiNi_{0.92}Co_{0.06}Mn_{0.02}O_2$	LZACO	66 (0.33 C)	30	5	95% (69 cycles)	This work
					90% (119 cycles)	
					80% (215 cycles)	
$LiCoO_2$	$Li_{1.625}Al_{0.375}Zr_{0.625}Cl_{5.25}$	~70 (0.5 C)	30	9.62	~58.71% (282 cycles)	(79)
$LiCoO_2$	Li_2ZrCl_6	~70 (0.5 C)	30	9.62	~48.74% (109 cycles)	(79)
$LiNi_{0.83}Co_{0.11}Mn_{0.06}O_2$	Li_3InCl_6	n/a	80	2	65% (50 cycles)	(35)
				10	93% (50 cycles)	(35)
$LiNi_{0.5}Co_{0.2}Mn_{0.3}O_2$	$Li_{0.388}Ta_{0.238}La_{0.475}Cl_3$	~66 (0.44 C)	30	~2	81.6% (100 cycles)	(11)

Supplementary Table 14. Parameters used for the phase-field simulation.

Parameter		Value
Thickness of the solid-electrolyte layer		137.9 μm
Thickness of the composite positive electrode layer		58.6 μm
Mass fraction of scNCM92 in the composite positive electrode		75%
Mass fraction of the solid electrolyte in the composite positive electrode		25%
scNCM92	Hardness	12 GPa
	Young′s modulus	91.04 GPa
	Poisson's ratio	0.3
LZCO	Hardness	2.07 GPa
	Young′s modulus	7.92 GPa
	Poisson's ratio	0.18
LZACO	Hardness	0.22 GPa
	Young′s modulus	1.41 GPa
	Poisson's ratio	0.18
Energy release rate of the solid electrolyte		1 J m^{-2}
Stacking pressure		190 MPa
